# Correlation between soluble klotho and chronic kidney disease–mineral and bone disorder in chronic kidney disease: a meta-analysis

**DOI:** 10.1038/s41598-024-54812-4

**Published:** 2024-02-23

**Authors:** Zhongyu Fan, Xuejiao Wei, Xiaoyu Zhu, Kun Yang, Ling Tian, Yujun Du, Liming Yang

**Affiliations:** Department of Nephrology, The First Hospital of Jilin University, Changchun, China

**Keywords:** Soluble Klotho, Chronic kidney disease–mineral and bone disorder, Chronic kidney disease, Mineral metabolism, Vascular calcification, Biomarkers, Diseases, Medical research, Nephrology, Risk factors, Urology

## Abstract

We conducted a systematic search across medical databases, including PubMed, Web of Science, EMBASE, and Cochrane Library, up to March 2023. A total of 1944 subjects or individuals from 17 studies were included in our final analysis. The correlation coefficient (r) between sKlotho and calcium was [0.14, (0.02, 0.26)], and a moderate heterogeneity was observed (I^2^ = 66%, *P* < 0.05). The correlation coefficient (r) between Klotho and serum phosphate was [− 0.21, (− 0.37, − 0.04)], with apparent heterogeneity (I^2^ = 84%, *P* < 0.05). The correlation coefficient (r) between sKlotho and parathyroid hormone and vascular calcification was [− 0.23,(− 0.29, − 0.17); − 0.15, (− 0.23, − 0.08)], with no significant heterogeneity among the studies. (I^2^ = 40%, *P* < 0.05; I^2^ = 30%, *P* < 0.05). A significant correlation exists between low sKlotho levels and an increased risk of CKD–MBD in patients with CKD. According to the findings, sKlotho may play a role in alleviating CKD–MBD by lowering phosphorus and parathyroid hormone levels, regulating calcium levels, and suppressing vascular calcification. As analysis showed that sKlotho has an important impact on the pathogenesis and progression of CKD–MBD in CKD patients. Nonetheless, further comprehensive and high-quality studies are needed to validate our conclusions.

## Introduction

Chronic kidney disease (CKD), defined as renal impairment lasting at least three months, is a growing global-scale health issue^1^ .Approximately 5–10% of the global population is affected by CKD^2^, which silently and asymptomatically develops in the early stages causing complications and ultimately leading to end-stage renal disease (ESRD). It necessitates renal replacement therapies such as dialysis or kidney transplantation for survival^3^. Mineral and bone disorders, cardiovascular disease, fluid imbalances and anemia are the most common complications of CKD^4^. Evidence of mineral metabolism disorders and bone abnormalities become apparent even in the early stages of CKD^5^.

Chronic kidney disease–mineral and bone disorder(CKD–MBD) is one of the severe complications associated with CKD^6^. CKD–MBD, a systemic disease caused by CKD, is characterized by disturbances in mineral and bone metabolism^7^. Its manifestation includes abnormalities in serum calcium (Ca), phosphorus(P), parathyroid hormone (PTH), as well as the occurrence of vascular or other soft tissue calcification^8,9^. CKD–MBD has been found to be a predominant contributor to decreased quality of life, increased mortality and morbidity in patients with CKD, as previous studies have shown^10^.

Klotho was initially characterized as an antiaging protein and it is prominently expressed in the tubular epithelial cells of the kidney^11^. Overexpression of klotho gene offers protection against a variety of pathological phenotypes, most notably in renal disease^12^. Klotho proteins encompass the following subfamilies: α-Klotho, β-Klotho, and γ-Klotho. α-Klotho can be found in kidneys and parathyroid glands. β-Klotho is expressed in liver and adipose tissue;The γ-Klotho is expressed in the eye, connective tissue^13^. The human Klotho protein exists in two forms: membrane-bound Klotho (mKlotho) and soluble Klotho (sKlotho)^13^. mKlotho is a transmembrane protein with a single-pass structure comprising 1,012 amino acids^14^. By anchoring proteases, the extracellular domain (Kl1 and Kl2) of mKlotho can be shed constitutively, generating sKlotho that is present in the cerebrospinal fluid, blood, and urine^13^. Experimental evidence has demonstrated that klotho, particularly sKlotho, exerts regulatory effects on phosphate and calcium metabolism, confers protection against oxidative stress, suppresses apoptosis, and possesses anti-inflammatory in the kidney^15,16^. Consequently, Klotho deficiency is considered to be a prevalent characteristic of kidney disease and plays an essential role in its pathogenesis and progression, including CKD and associated complications^17^. Timely detection and prevention of CKD–MBD are paramount for releasing the burden on CKD patients.

Accumulating evidence indicates that Klotho may have potential as an early biomarker for CKD–MBD^17–19^. Accordingly, by collecting relevant literature data and conducting a meta-analysis, we can gain a more comprehensive understanding to verify whether Klotho is a feasible biomarker for the diagnosis and prediction of CKD–MBD progression.

## Methods and materials

### Study design

The study has been registered in the International Database of Prospective Systematic Reviews (Registration No. CRD42022385019) and conducted following the Preferred Reporting Items for Systematic Reviews and Meta-Analyses (PRISMA)^20^.

### Data sources and search strategy

The electronic databases PubMed, Web of Science, EMBASE and Cochrane Library were comprehensively queried for pertinent literature from their inception until March 11, 2023. Each publication's bibliography was also perused to discover any additional material related to this subject.

If necessary, we will contact the authors who conducted the investigation to obtain more information regarding the eligibility criteria in order to respond to any inquiries. The Supplemental 1 provides the search terms and a detailed search approach.

### Study inclusion and exclusion criteria

Inclusion criteria: (1) Participants were adults aged at least 18 years; (2) The study type was cohort or observational; (3) The study included complete data information; (4) The publication language is English; (5) Include at least one outcome relevant to the study of interest.

Exclusion criteria: (1) Incomplete study data or statistical errors; (2) Duplicate studies; (3) Nonclinical research, including in vivo or in vitro experimental; (4) Overlapping or duplicated research populations; (5) Case reports, abstracts, reviews, conference summaries and comments; (6) Study aims to investigate the correlation between renal klotho expression, including mRNA and protein levels, and other relevant parameters; (7) Special participants, such as patients who have undergone kidney transplantation. Exclude patients with diabetic nephropathy; (8) The research design is not standardized.

### Data extraction and quality assessment

Using a standardized form, two independent investigators (Zhongyu Fan and Xuejiao Wei) extracted the data. The following data were extracted: First author, year of publication, location, age, research index, sample size, study type, disease models and correlation coefficient (Pearson or Spearman). We transformed the published Pearson correlation coefficient into Spearman correlation coefficient in order to better use the data because the latter are unaffected by the logarithmic modification. In addition, by applying the Fisher’s transformation, the correlation coefficients are converted into approximately normally distributed Z-values^21^. The standard deviation (standard error, SE) of Z is then calculated, followed by the inverse Fisher’s transformation of the Z-values to obtain the correlation coefficient and confidence intervals (CI). If data is unavailable or incomplete, the corresponding authors will be contacted to obtain the relevant data. The disagreement in data extraction were resolved by consulting a third reviewer. Two authors (Xiaoyu Zhu and Kun Yang) evaluated the research quality and risk of bias using the Newcastle–Ottawa Scale (NOS)^22^. Studies that received a score of 7 or higher were deemed to have a low risk of bias and were classified as high-quality studies.

### Meta-analysis and statistical analysis

Data analysis was conducted using Review Manager 5.4 software (Cochrane Collaboration, Copenhagen, Denmark). When I^2^ < 50%, a fixed-effect model was employed. When I^2^ ≥ 50%, a random-effect model was used^23^. In the presence of significant heterogeneity, sensitivity and subgroup analyses were conducted to examine the sources of heterogeneity. A sensitivity analysis was conducted, excluding each study sequentially, to ensure the consistency of the outcomes. We visually examined the probability of publication bias by using funnel plots. A two-tailed test with *P* < 0.05 was considered statistically significant.

## Results

After searching PubMed, Embase, Cochrane Library and Web of Science, a total of 2382 publications that were relevant to the topic were selected (PubMed: n = 535; Embase: n = 648; Cochrane Library: n = 71; Web of Science: n = 1128).

**Figure 1:**
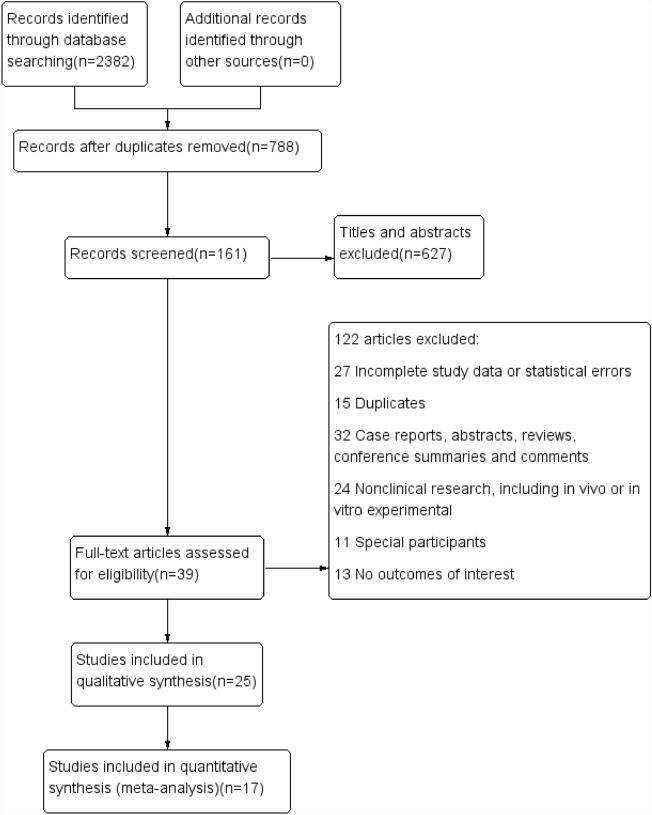
Screening procedures for the study.

**Figure 2:**
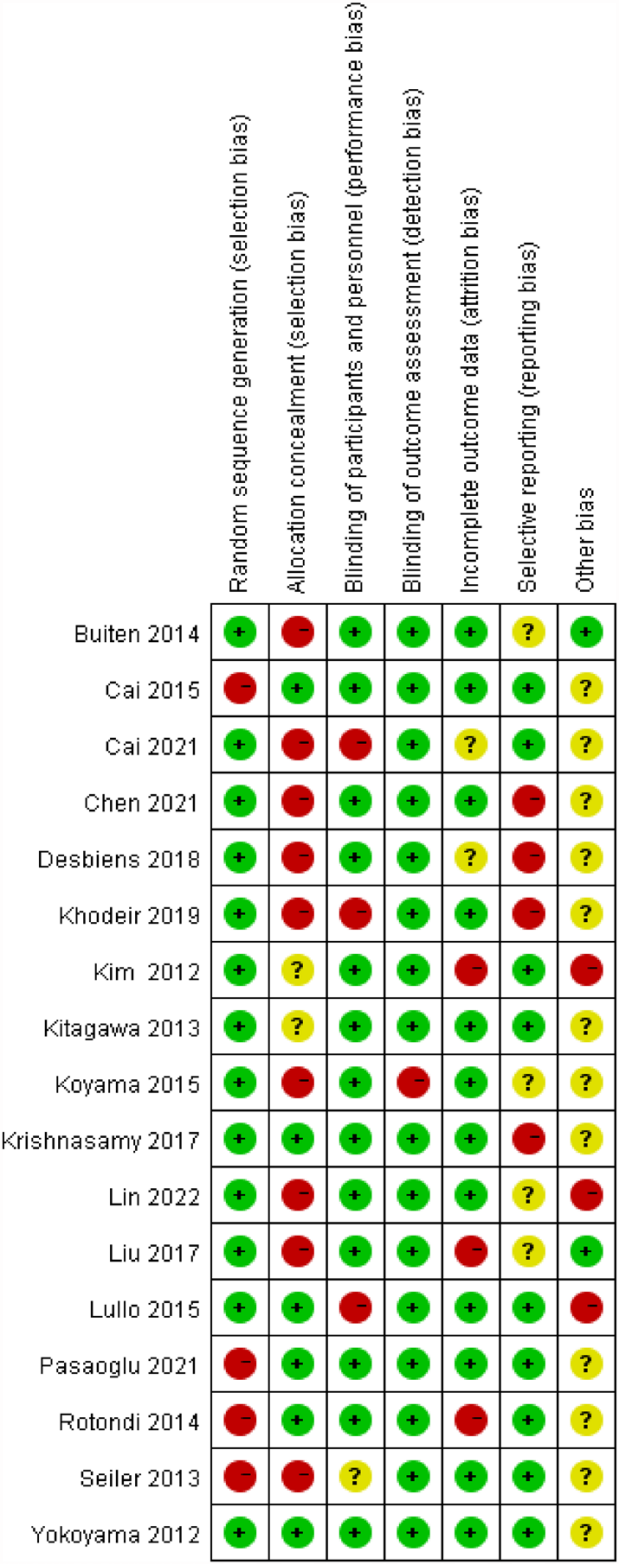
Inclusion of literature quality assessment bias risk summary figure.

In these studies, a total of 788 duplicates were removed. Through screening titles and abstracts, a total of 627 publications were excluded. After reading the full texts, an additional 122 articles were excluded due to various reasons, resulting in a final selection of 17 studies for our meta-analysis. The procedure for selecting articles is showed in Fig. 1. The risk of bias in included literature is displayed in Fig. 2.

Among them, eight article or studies[24–31]with a combined total of 920 participants published data on Klotho and calcium. With a total of 1018 participants, data on Klotho and phosphorus were presented in 8 publications^24–27,29–32^. Eight articles^24,25,27,29,32–35^ with a total of 1280 participants presented data on Klotho and parathyroid hormone. Data on Klotho and vascular calcification were provided in 6 articles^27,34,36–39^, with a total of 651 participants. In addition to the ten articles from Asia, there was one article from North America, four articles from Europe, one article from Africa, and one article from Oceania. Both males and females were involved in each of the 17 studies (Table 1).

**Table 1:** Characteristics of the studies included.

References	Country	Study design	Assay utilization	N	Average age(years)	Disease model	Index	Correlation coefficient	NOS scores
Lin^39^	China	cross-sectional study	IBL ELISA	140	52.15	MHD	VC	− 0.72	6
Cai^37^	China	cross-sectional study	IBL ELISA	128	58.29	MHD	VC	− 0.213	7
Pasaoglu^29^	Turkey	cross-sectional study	IBL ELISA	60	48.22	MHD	Ca P PTH	0.088 ∗ 0.127 ∗ − 0.084 ∗	8
Chen^32^	China	cross-sectional study	IBL ELISA	180	58	CKD	P PTH	− 0.487 ∗ − 0.378 ∗	6
Khodeir^26^	Egypt	cross-sectional study	IBL ELISA	50	55.53	CKD	Ca P	0.449 ∗ − 0.490 ∗	6
Desbiens^25^	Canada	cross-sectional study	IBL ELISA	130	72	MHD	Ca P PTH	0.028 ∗ − 0.062 ∗ − 0.073 ∗	7
Krishnasamy(65)	Australia	cross-sectional study	IBL ELISA	40	62.9	CKD	VC	− 0.36	8
Liu(66)	China	cross-sectional study	IBL ELISA	112	45.3	CKD	Ca	0.302 ∗	8
Koyama^27^	Japan	cross-sectional study	IBL ELISA	52	78.2	CKD	Ca P PTH	0.0596 ∗ − 0.285 ∗ − 0.239 ∗	7
Cai^36^	China	cross-sectional study	IBL ELISA	129	58.18	MHD	VC	− 0.214 ∗	8
Di Lullo^38^	Italy	cross-sectional study	IBL ELISA	100	51	CKD	VC	− 0.208	9
Rotondi^30^	Italy	cross-sectional study	IBL ELISA	68	58	CKD	Ca P	0.3 − 0.28	6
Buiten^24^	The Netherlands	cross-sectional study	IBL ELISA	127	67	MHD	Ca P PTH	− 0.04 ∗ − 0.04 ∗ − 0.26 ∗	7
Kitagawa^34^	Japan	cross-sectional study	IBL ELISA	114	58	CKD	PTH VC	− 0.3034 ∗ 0.0245 ∗	9
Kim^33^	Korea	cross-sectional study	IBL ELISA	140	52.15	MHD	PTH	− 0.72	7
Seiler^31^	Germany	cross-sectional study	IBL ELISA	321	66.6	CKD	Ca P	0.01 ∗ − 0.06 ∗	6
Yokoyama^35^	Japan	cross-sectional study	IBL ELISA	53	58.6	MHD	PTH	− 0.08	9

∗ Spearman relation; CKD, chronic kidney disease; MHD, maintenance hemodialysis; Ca, calcium; P, phosphate; PTH, parathyroid hormone; VC, vascular calcification. IBL, Immuno-Biologic Laboratories Co.

### The association between sKlotho and calcium

**Figure 3:**
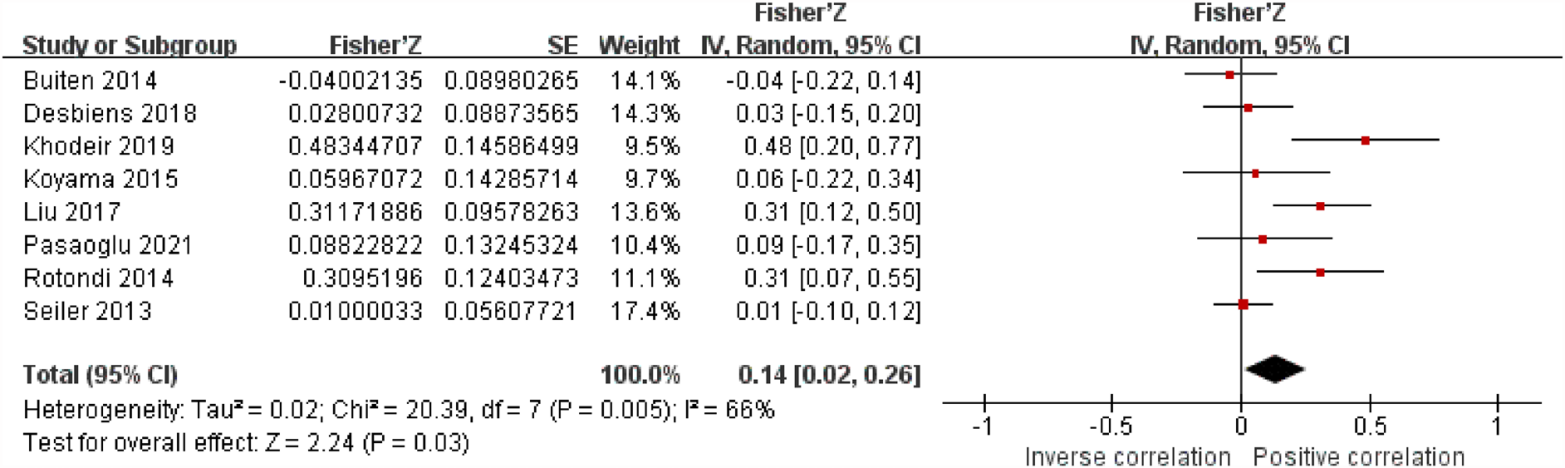
Forest plots of the summary r with effect estimate pooled r (95% CI) for the association between sKlotho level and Ca.

Figure 3 presents a meta-analysis of the correlation between sKlotho and Ca. Seven included studies demonstrated a positive correlation between sKlotho and Ca, while one study showed a negative correlation. There was moderate heterogeneity among the eight studies (I^2^ = 66%, *P* < 0.05), so a random-effect meta-analysis was conducted, and the pooled correlation coefficient(r) and its 95% CI were [0.14, (0.02,0.26)], indicating a significant positive correlation between sKlotho and Ca.

Sensitivity analysis by removing any study from this study did not affect the results, suggesting the stability and reliability of the random-effects calculations.

As shown in the funnel plot (Supplemental Fig. 1), the distribution is asymmetric, indicating the presence of publication bias.

To further investigate the underlying causes of the considerable heterogeneity observed in these studies, subgroup meta-analyses were performed, stratifying the analysis based on disease model (Pre-dialysis or Dialysis), average age (≥ 60 years or < 60 years), sample size (N ≥ 100 or N < 100), and study quality (score ≥ 7 or < 7).The results of the subgroup meta-analyses are shown in Table 2 and Supplemental Figs. 2a-d. In all subgroups, the positive correlation remained significant. Subgroup age exhibited statistical apparent heterogeneity (I^2^ = 93.7%, *P* < 0.05). Therefore, age was considered as potential source of heterogeneity among the studies .

**Table 2:** Subgroup analysis results of sKlotho level and Ca.

Subgroup	Studies	Effect estimate pooled r (95% CI)	Heterogeneity within each group	Heterogeneity between subgroup
Disease models	8	0.14 [0.02, 0.26]		*P* = 0.06, I^2^ = 71.8%
Pre-dialysis	5	0.22 [0.03, 0.41]	*P* = 0.02, I^2^ = 75%	
Dialysis	3	0.01 [− 0,10, 0.12]	*P* = 0.84, I^2^ = 0%	
Age	8	0.10 [0.03, 0.16]		*P* < 0.0001,I^2^ = 93.7%
Age ≥ 6o years	4	0.01 [− 0.07, 0.09]	*P* = 0.85, I^2^ = 0%	
Age < 60y ears	4	0.29 [0.18, 0.41]	*P* < 0.00001,I^2^ = 28%	
Sample size	8	0.14 [0.02, 0.26]		*P* = 0.17, I^2^ = 47.3%
N ≥ 100	4	0.07 [− 0.07, 0.21]	*P* = 0.32, I^2^ = 67%	
N < 100	4	0.23 [0.05, 0.42]	*P* = 0.01, I^2^ = 50%	
Study quality	8	0.14 [0.02, 0.26]		*P* = 0.35, I^2^ = 0%
High-quality study(≥ 7 stars)	5	0.09 [− 0.04, 0.22]	*P* = 0.18, I^2^ = 50%	
Low-quality study(< 7 stars)	3	0.25[− 0.05, 0.54]	*P* = 0.11, I^2^ = 84%	

### The association between sKlotho and phosphate

**Figure 4:**
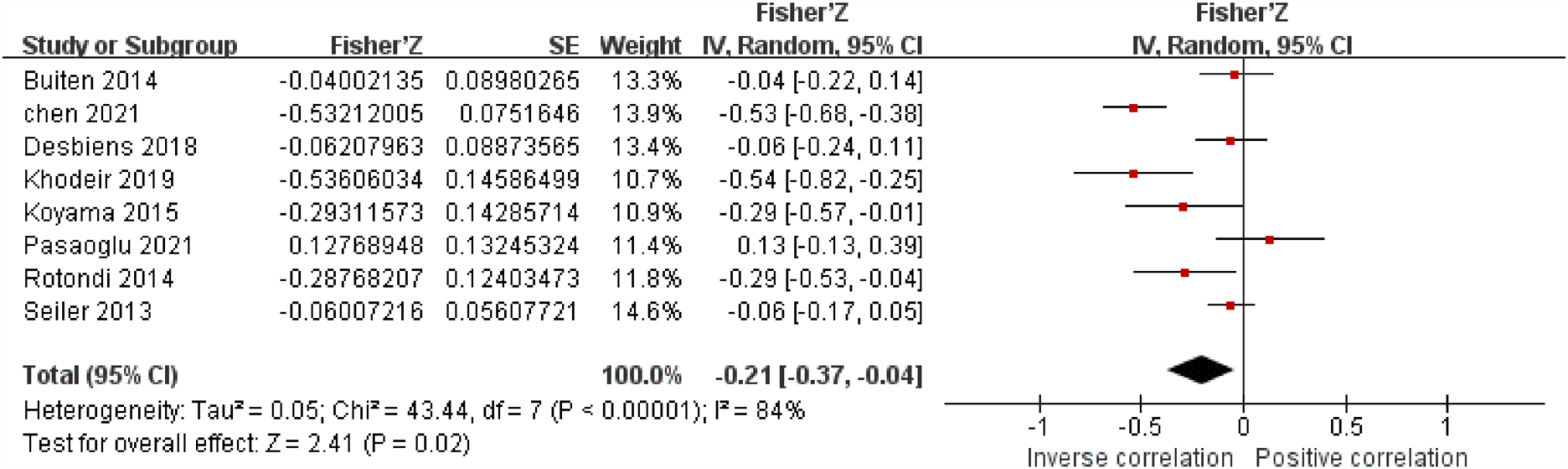
Forest plots of the summary r with effect estimate pooled r (95% CI) for the association between sKlotho level and P.

Eight studies were evaluated to assess the relationship between sklotho and phosphate. Seven studies found a positive correlation between sklotho and P, whereas one found an inverse correlation. The studies were found to have a high degree of heterogeneity (I^2^ = 84%, *P* < 0.05). So a random-effect meta-analysis was performed, and the pooled correlation coefficient(r) and its 95% CI were [-0.21,(-0.37,-0.04)], demonstrating a remarkable negative connection between sKlotho and P (Fig. 4).

Sensitivity analysis conducted on these eight articles showed that none of them significantly influenced the results of the meta-analysis, indicating strong stability using random-effects calculations.

The symmetry observed in the funnel plot suggests without presence of publication bias in the literature included in this study. (Supplemental Fig. 3).

Furthermore, the subgroup analysis results demonstrated that disease model possible contributors to the observed heterogeneity (I^2^ = 82.7%, *P* < 0.05) (Table 3 and Supplemental Figs. 4a-d).

**Table 3:** Subgroup analysis results of sKlotho level and P.

Subgroup	Studies	Effect estimate pooled r (95% CI)	Heterogeneity within each group	Heterogeneity between subgroup
Disease models	8	− 0.21 [− 0.37, − 0.04]		*P* = 0.02, I^2^ = 82.7%
Pre-dialysis	5	− 0.33 [− 0.57, − 0.10]	*P* = 0.005, I^2^ = 86%	
Dialysis	3	− 0.02 [− 0,13, 0.09]	*P* = 0.75, I^2^ = 0%	
Age	8	− 0.21 [− 0.37, − 0.04]		*P* = 0.13, I^2^ = 56.7%
Age ≥ 60 years	4	− 0.07 [− 0.15, 0.00]	*P* = 0.06, I^2^ = 0%	
Age < 60y ears	4	− 0.31 [− 0.61, − 0.02]	*P* = 0.04, I^2^ = 85%	
Sample size	8	− 0.21 [− 0.37, − 0.04]		*P* = 0.71, I^2^ = 0%
N ≥ 100	4	− 0.17 [− 0.41, 0.06]	*P* = 0.15, I^2^ = 90%	
N < 100	4	− 0.24 [− 0.51, 0.02]	*P* = 0.07, I^2^ = 75%	
Study quality	8	− 0.21 [− 0.37, − 0.04]		*P* = 0.07, I^2^ = 69.8%
High-quality study(≥ 7 stars)	4	− 0.06 [− 0.19, 0.08]	*P* = 0.39, I^2^ = 36%	
Low-quality study(< 7 stars)	4	− 0.34 [− 0.62, − 0.07]	*P* = 0.02, I^2^ = 90%	

### The association between sKlotho and parathyroid hormone

**Figure 5:**
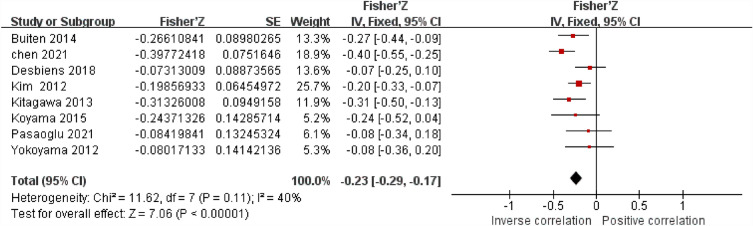
Forest plots of the summary r with effect estimate pooled r (95% CI) for the association between sKlotho level and PTH.

The connection between sKlotho and blood levels of PTH was investigated in eight different studies. All eight literatures showed a negative correlation. The studies' heterogeneity was found to be modest (I^2^ = 40%, *P* < 0.05). Using fixed-effects meta-analysis, the pooled correlation coefficient(r) and its 95% CI were [-0.23, (-0.29,-0.17)] (Fig. 5).

**Figure 6:**
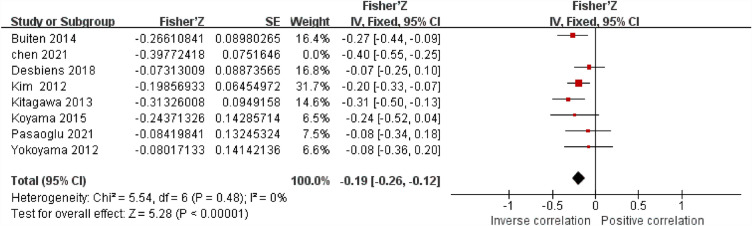
Forest plots of the summary r with effect estimate pooled r (95% CI) for the association between sKlotho level and PTH after removing Chen’s study.

Sensitivity analysis showed that the study by Chen et al. has a significant effect on heterogeneity (I^2^ = 0, *P* < 0.05), but taking this study out didn't change the total result [-0.19 (-0.26, − 0.12)] (Fig. 6).

As evidenced by the funnel plot, there is an asymmetrical distribution, suggesting the existence of publication bias. (Supplemental Fig. 5).

There was no signifcant heterogeneity between subgroups in analyses of disease model, average age, sample size and study quality (*P* > 0.05) (Table 4 and Supplemental Fig. 6a-d).

**Table 4:** Subgroup analysis results of sKlotho level and PTH.

Subgroup	Studies	Effect estimate pooled r (95% CI)	Heterogeneity within each group	Heterogeneity between subgroup
Disease models	9	− 0.18 [− 0.29, − 0.06]		*P* = 0.50, I^2^ = 0%
Pre-dialysis	4	− 0.14 [− 0.25, − 0.04]	*P* = 0.007, I^2^ = 0%	
Dialysis	5	− 0.21 [− 0,40, − 0.03]	*P* = 0.02, I^2^ = 85%	
Age	9	− 0.18 [− 0.29, − 0.06]		*P* = 0.22, I^2^ = 34.8%
Age ≥ 60 years	4	− 0.11 [− 0.28, 0.05]	*P* = 0.17, I^2^ = 82%	
Age < 60y ears	5	− 0.24 [− 0.36, − 0.12]	*P* < 0.0001, I^2^ = 50%	
Sample size	9	− 0.18 [− 0.29, − 0.06]		*P* = 0.57, I^2^ = 0%
N ≥ 100	6	− 0.20 [− 0.34, − 0.05]	*P* = 0.009, I^2^ = 83%	
N < 100	3	− 0.13 [− 0.29, 0.02]	*P* = 0.10, I^2^ = 0%	
Study quality	9	− 0.18 [− 0.29, − 0.06]		*P* = 0.77, I^2^ = 0%
High-quality study(≥ 7 stars)	5	− 0.20 [− 0.29, − 0.12]	*P* < 0.00001, I^2^ = 0%	
Low-quality study (< 7 stars)	4	− 0.17 [− 0.39, 0.05]	*P* = 0.13, I^2^ = 88%	

### The association between sKlotho and vascular calcification

**Figure 7:**
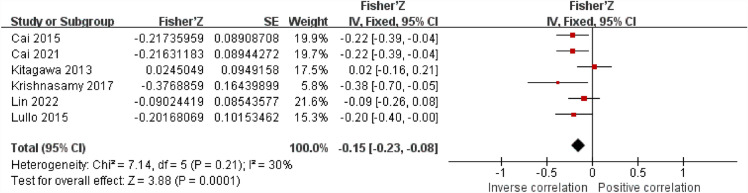
Forest plots of the summary r with effect estimate pooled r (95% CI) for the association between sKlotho level and VC.

There are six articles discussing the connection between sKlotho level and vascular calcification. Five studies discovered an inverse correlation between klotho and correlation, while one investigation indicated a positive correlation. The results of the meta-analysis reveal that there is inconspicuous heterogeneity among the studies using the fixed effect model (I^2^ = 30%, *P* < 0.05). The pooled correlation coefficient(r) and its 95% CI were [-0.15, (-0.23, − 0.08)] (Fig. 7).

Strong stability in this study is indicated by the sensitivity analysis done on these six publications, which revealed that none of them significantly affected the findings of the meta-analysis.

The identified symmetrical pattern in the funnel plot suggests no publication bias remain in the contained literature (Supplemental Fig. 7).

## Discussion

The kidney, and more specifically the distal tubules of the kidney, have a high level of sklotho expression^40,41^. sKlotho acts as a co-receptor for fibroblast growth factor 23 (FGF23)^42^. sKlotho plays an essential role in various essential physiological processes within the human body, including the regulation of calcium and phosphorus metabolism^14,43^. Research has demonstrated that the regulatory effects of sKlotho are primarily achieved through its interaction with target molecules^44^. Abnormally decreased levels of sKlotho have been associated with disturbances in serum calcium and phosphorus metabolism in patients^45,46^. Studies have also confirmed that klotho gene knockout mice exhibit abnormal urinary phosphorus excretion and hyperparathyroidism, leading to hypophosphatemia, osteoporosis, and osteomalacia^47^. Thus, Klotho is closely related to calcium and phosphorus metabolism, PTH synthesis and secretion, and vascular calcification^44,48^.

To the best of our knowledge, our study is the first to conduct a meta-analysis on the correlation between sKlotho levels and CKD–MBD in patients with CKD. In this article, we investigated the correlation between sklotho and calcium, phosphorus, parathyroid hormone and vascular calcification. Positive correlation between sKlotho and calcium, as well as negative correlation with phosphorus, parathyroid hormone, and vascular calcification were observed, all of which were statistically significant. Although moderate heterogeneity was observed in the study of the correlation between sklotho and calcium and phosphorus (I^2^ = 57%, *P* < 0.05; I^2^ = 57%, *P* < 0.05), the correlation still exists in sensitivity analysis, indicating that the conclusion obtained might be considered relatively reliable.

Acting as an autocrine or paracrine enzyme, Klotho modulates the process through downregulation of the renal sodium phosphate cotransporter NaPi-2a expression^46^. This action leads to a decrease in urine phosphorus reabsorption, effectively countering hyperphosphatemia^49^. The soluble form of Klotho, acting independently, enhances calcium reabsorption while diminishing calcium loss in the kidneys through the regulation of calcium-selective channels^50,51^. Additionally, it has been observed that Klotho increases calcium absorption in urine by stabilizing the TRPV5 calcium channel, regardless of the presence of FGF23^52^. The analysis conducted in our study unveiled a significant discovery: a negative correlation between klotho and serum phosphorus, along with a positive correlation between klotho and serum calcium. These correlations were statistically significant, consistent with the aforementioned research findings.

The presence of sKlotho has been identified in parathyroid tissue, and research has revealed that the excessive depletion of the Klotho-FGF receptor complex in patients with end-stage renal illness is linked to the overproduction of PTH and secondary hyperparathyroidism (SHPT)^53,54^. This investigation observed a negative correlation between sKlotho and PTH, suggesting that sKlotho exerts an inhibitory influence on PTH production.

In vitro studies have demonstrated that the presence of sKlotho impedes the process of sodium-dependent phosphate uptake and mineralization, often triggered by hyperphosphate^55^. According to recent research, people with MHD who have high levels of sKlotho had a 61% lower risk of cardiovascular events and cardiovascular mortality when compared to those who have low levels of sKlotho^56^. sKlotho suppresses vascular calcification (VC) by obstructing the Wnt/β-catenin signaling pathway and preventing the development of vascular osteoblasts^57^. The absence of Klotho has been associated with the occurrence of soft tissue calcification in patients with CKD^58^. sKlotho has also been found to enhance phosphaturia, preserve glomerular filtration, and directly inhibit phosphate absorption by vascular smooth muscle, thereby improving vascular calcification^46,50,59^. According to a prior study, patients with higher levels of sKlotho showed a decreased risk of serum creatinine doubling^28^.

Reportedly, sKlotho directly modulates renal calcium and phosphorus excretion, triggers the activation of the ERK1/2-SGK1-NHERF-1-NaPi-2a pathway upstream, and engages in systemic mineral homeostasis through 1-αhydroxylase activity^60^. sklotho inhibits parathyroid glands' production of PTH, primarily via the well-known Klotho-dependent pathway of mitogen-activated protein kinases (MAPK) activation, or, less well-known, via the activation of the NFAT cascade by phospholipase C gamma (PLCγ)^61^.

According to the results presented above, it becomes evident that sKlotho has the ability to lower serum phosphorus and PTH levels, regulate blood calcium levels, and inhibit vascular calcification through various mechanisms, regardless of the presence or absence of FGF23. The meta-analysis reached a unanimous conclusion, which is consistent with the presented findings. Soluble klotho is merely one of numerous factors impacting mineral metabolism. Many other variables, including specific medications, involvement in inflammation, and dietary factors, influence serum phosphorus, parathyroid hormone, and calcium levels. Our analytical approach cannot comprehensively eliminate these confounding variables.

In the light of the subgroup analysis obtained, we identified that the study's heterogeneity concerning sklotho and serum calcium primarily stems from age. In the subgroup analysis investigating the correlation between sklotho and serum phosphorus, we noted that both age and disease models serve as the main sources of heterogeneity. Hence, the presence of heterogeneity might be attributable to factors such as age and the patients' dialysis status. Not surprisingly, a correlation exists between Klotho and lifespan, as supported by research indicating a decline in sKlotho levels with advancing age^14,62^. This suggests that the expression of sKlotho is regulated by the aging process. Earlier studies have established a significant link between reduced sKlotho levels and unfavorable outcomes among those undergoing dialysis^63^. The impact of sKlotho levels on clinical outcomes could potentially diminish as individuals age. A prospective association between sKlotho and the aging process emerges. Existing research has indicated that chronic renal failure profoundly affects gene expression and kidney function in MHD patients^64^. Taken together, it is plausible that age and dialysis status may act as prominent confounding variables and contributors to heterogeneity.

This meta-analysis has certain limitations. Firstly, the sample sizes of participants in several of the included studies is relatively small, despite the high overall number of patients engaged in this meta-analysis. Consequently, even though we applied a random-effects model for our research, it may result in an underestimation of our overall results. For all these reasons, further research is necessary to validate our findings. Secondly, this analysis relies on published studies, which often report positive and significant outcomes. Studies with negative or non-significant results are more prone to rejection. Additionally, certain results related to our analysis are still in the process of being accepted, potentially leading to publication bias. Thirdly, some of the studies included in our analysis only presented relevant indicators as positive or negative correlations, without providing r-values or the raw data required to calculate correlation coefficients. Fourthly, some of the outcomes exhibit heterogeneity, and although this can be partially reduced through subgroup or sensitivity analyses, potential confounding factors may still exist due to differences in the study populations and sklotho measurement methods. Nevertheless, since the number of included studies is limited, more detailed subgroup analyses cannot be carried out, which could influence the precision and usefulness of the results.

In summary, the findings of our study point to a robust association between sKlotho and CKD–MBD in patients with CKD, leading to a notable alleviation of mineral metabolism challenges within this population. However, it is crucial to recognize that this meta-analysis is subject to certain limitations. The outcomes of this study offer a positive outlook on the potential utility of sKlotho as a feasible biomarker for patients with CKD–MBD. Nevertheless, further comprehensive and rigorous investigations are imperative to validate our findings.

### Supplementary Information

Supplementary Information.

## Data Availability

The supporting data of this study can be obtained from the corresponding author upon reasonable request.
