# Supplementary material for: Correlation between soluble klotho and chronic kidney disease–mineral and bone disorder in chronic kidney disease: a meta-analysis
Source: Sci Rep. 2024 Feb 23;14:4477. doi: 10.1038/s41598-024-54812-4 (PMC10891172; doi:10.1038/s41598-024-54812-4)

**Supplemental 1**. Search terms and a detailed search approach.

**Pubmed**：

(((Chronic Kidney Disease-Mineral and Bone Disorder[MeSH Terms]) OR (Chronic kidney diseasemineral bone disorders[Title/Abstract] OR CKD-MBD[Title/Abstract]OR CKD-mineral and bone disorder[Title/Abstract] bone diseases[Title/Abstract] OR bone diseases metabolic[Title/Abstract] OR bone demineralization[Title/Abstract] OR renal osteodystrophy[Title/Abstract] OR hyperparathyroidism[Title/Abstract] OR secondary hyperparathyroidism[Title/Abstract] OR hyperphosphatemia[Title/Abstract] OR calcinosis[Title/Abstract] OR vascular calcification[Title/Abstract] OR soft tissue calcification[Title/Abstract] OR phosphorus[Title/Abstract] OR phosphate[Title/Abstract] OR calcium[Title/Abstract] OR Parathyroid hormone[Title/Abstract] OR PTH[Title/Abstract] OR SHPT[Title/Abstract])) AND ((Klotho protein[MeSH Terms]) OR (soluble klotho[Title/Abstract] OR secreted klotho[Title/Abstract] OR skloth[Title/Abstract] OR alpha klotho[Title/Abstract] OR α-Klotho[Title/Abstract] OR kl[Title/Abstract] OR skl[Title/Abstract] OR αKL[Title/Abstract] OR klotho[Title/Abstract] OR KL protein[Title/Abstract] OR alpha-Klotho protein[Title/Abstract]))) AND ((chronic kidney disease[MeSH Terms]) OR (CKD[Title/Abstract] OR chronic renal insufficiency[Title/Abstract] OR chronic renal failure[Title/Abstract] OR chronic kidney insufficiency[Title/Abstract] OR chronic nephropathy[Title/Abstract] OR chronic renal disease[Title/Abstract] OR chronic kidney failure[Title/Abstract] OR end stage renal disease[Title/Abstract] OR end stage kidney disease[Title/Abstract] OR ESRD[Title/Abstract] OR ESKD[Title/Abstract] OR pre-dialysis[Title/Abstract] OR dialysis[Title/Abstract] OR renal dialysis[Title/Abstract] OR uremic[Title/Abstract] OR uremia[Title/Abstract] OR hemodialysis[Title/Abstract] OR HD[Title/Abstract] OR peritoneal dialysis[Title/Abstract] OR PD[Title/Abstract]))

**Cochrane Library：**

#1 MeSH descriptor: [Chronic Kidney Disease-Mineral and Bone Disorder] explode all trees

#2 (bone diseases OR bone diseases metabolic OR bone demineralization OR renal osteodystrophy OR hyperparathyroidism OR secondary hyperparathyroidism OR hyperphosphatemia OR calcinosis OR vascular calcification OR soft tissue calcification OR phosphorus OR phosphate OR calcium OR Parathyroid hormone OR PTH OR SHPT):ti,ab,kw (Word variations have been searched)

#3 #1 or #2

#4 MeSH descriptor: [Klotho Proteins] explode all trees

#5 (soluble klotho or secreted klotho or skloth or alpha klotho or α-Klotho or kl or skl or αKL or klotho OR KL protein OR alpha-Klotho protein):ti,ab,kw (Word variations have been searched)

#6 #4 or #5

#7 MeSH descriptor: [Renal Insufficiency, Chronic] explode all trees

#8 (CKD or chronic renal insufficiency or chronic renal failure or chronic kidney insufficiency or chronic nephropathy or chronic renal disease or chronic kidney failure or end stage renal disease or end stage kidney disease or ESRD or ESKD or pre-dialysis or dialysis or renal dialysis or uremic or uremia or hemodialysis or HD or peritoneal dialysis or PD):ti,ab,kw (Word variations have been searched)

#9 #7 or #8

#10 #3 and #6 and #9

**Web of Science：**

1: (TS=(Chronic Kidney Disease-Mineral and Bone Disorder)) OR TS=(Chronic kidney diseasemineral bone disorders OR CKD-MBD OR CKD-mineral and bone disorder OR bone diseases OR bone diseases metabolic OR bone demineralization OR renal osteodystrophy OR hyperparathyroidism OR secondary hyperparathyroidism OR hyperphosphatemia OR calcinosis OR vascular calcification OR soft tissue calcification OR phosphorus OR phosphate OR calcium OR Parathyroid hormone OR PTH OR SHPT)

2: (TS=(Klotho protein)) OR TS=(soluble klotho or secreted klotho or skloth or alpha klotho or α-Klotho or kl or skl or αKL or klotho OR KL protein OR alpha-Klotho protein)

3: (TS=(chronic kidney disease)) OR TS=(CKD or chronic renal insufficiency or chronic renal failure or chronic kidney insufficiency or chronic nephropathy or chronic renal disease or chronic kidney failure or end stage renal disease or end stage kidney disease or ESRD or ESKD or pre-dialysis or dialysis or renal dialysis or uremic or uremia or hemodialysis or HD or peritoneal dialysis or PD)

**EMBASE：**

#10. #7 AND #8 AND #9

#9. #5 OR #6

#8. #3 OR #4

#7. #1 OR #2

#6. ((((((((((ckd OR chronic) AND renal AND insufficiency OR chronic) AND renal AND failure OR chronic) AND kidney AND insufficiency OR chronic) AND nephropathy OR chronic) AND renal AND disease OR chronic) AND kidney AND failure OR end) AND stage AND renal AND disease OR end) AND stage AND kidney AND disease OR esrd OR eskd OR 'pre dialysis' OR dialysis OR renal) AND dialysis OR uremic OR uremia OR hemodialysis OR hd OR peritoneal) AND dialysis OR pd

#5. chronic AND kidney AND disease

#4. ((((soluble AND klotho OR secreted) AND klotho OR skloth OR alpha) AND klotho OR 'α klotho' OR skl OR αkl OR klotho OR kl) AND protein OR 'alpha klotho') AND protein

#3. 'klotho protein'

#2. (chronic AND kidney AND diseasemineral AND bone AND disorders OR 'ckd mbd' OR 'ckd mineral') AND bone AND disorder OR bone) AND diseases OR bone) AND diseases AND metabolic OR bone) AND demineralization OR renal) AND osteodystrophy OR hyperparathyroidism OR secondary) AND hyperparathyroidism OR hyperphosphatemia OR calcinosis OR vascular) AND calcification OR soft) AND tissue AND calcification OR phosphorus OR phosphate OR calcium OR parathyroid) AND hormone OR pth OR shpt

#1. chronic AND ('kidney'/exp OR kidney) AND 'disease mineral' AND ('bone'/exp OR bone) AND ('disorder'/exp OR disorder)

**Supplemental Figure 1**. Funnel plot of sKlotho levels and Ca.


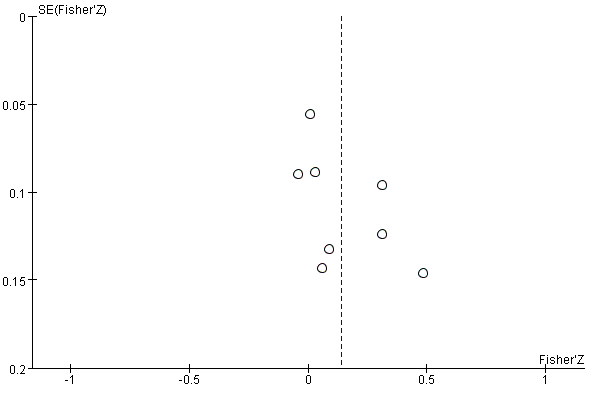


**Supplemental Figure 2a****.** Subgroup analysis results of sKlotho level and Ca on disease models.


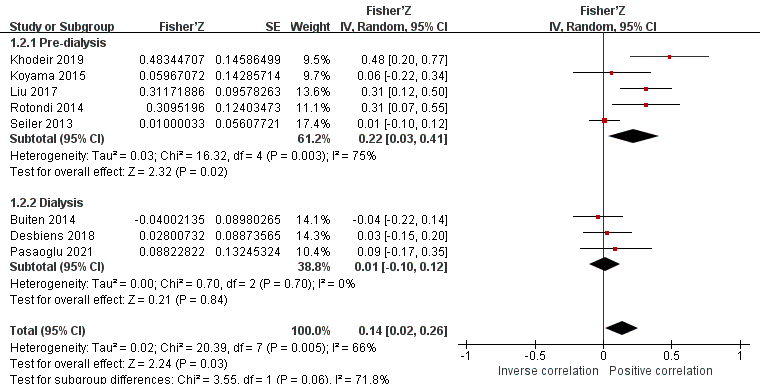


**Supplemental Figure 2b.** Subgroup analysis results of sKlotho level and Ca on age.


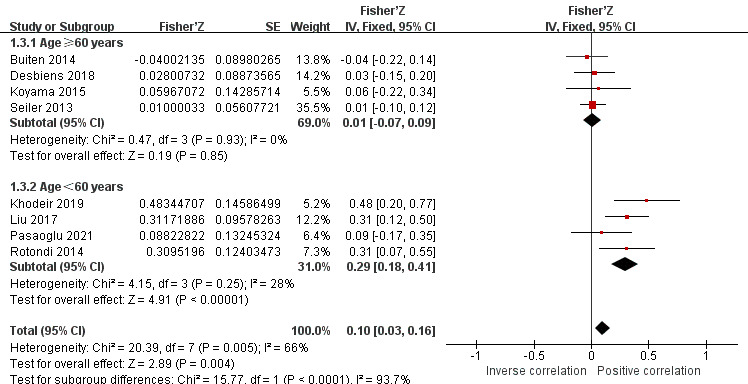


**Supplemental Figure 2c.** Subgroup analysis results of sKlotho level and Ca on sample size.


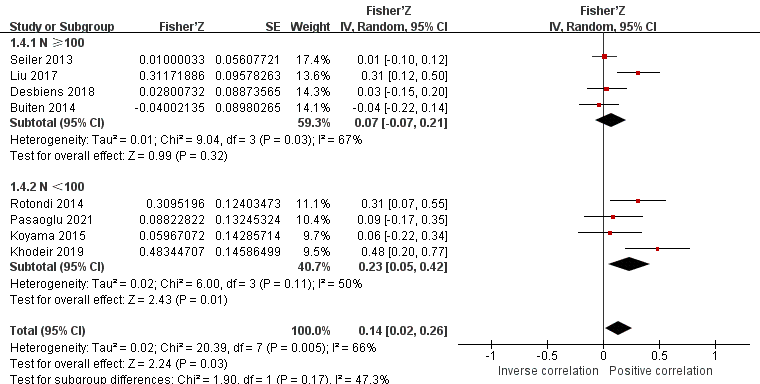


**Supplemental Figure 2d.** Subgroup analysis results of sKlotho level and Ca on study quality.


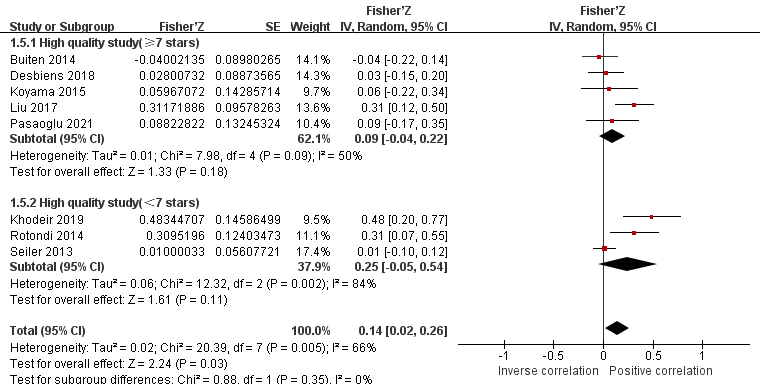


**Supplemental Figure 3.** Funnel plot of sKlotho levels and P.


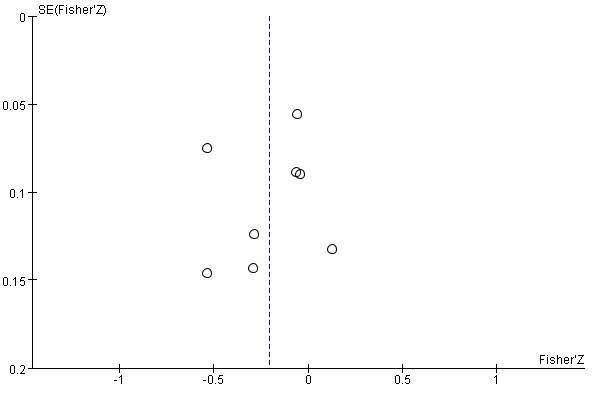


**Supplemental Figure 4a.** Subgroup analysis results of sKlotho level and P on disease models.


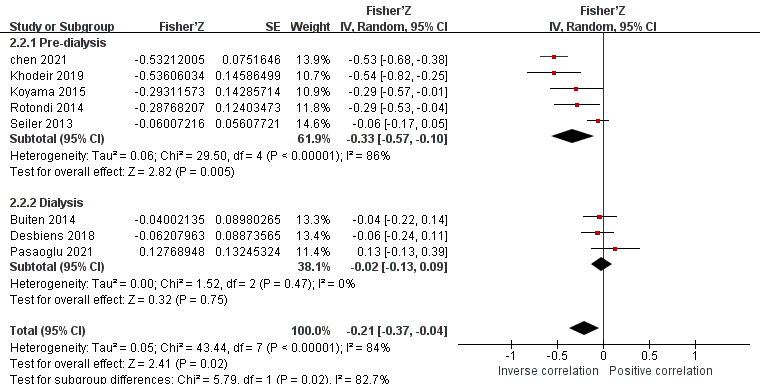


**Supplemental Figure 4b.** Subgroup analysis results of sKlotho level and P on age.


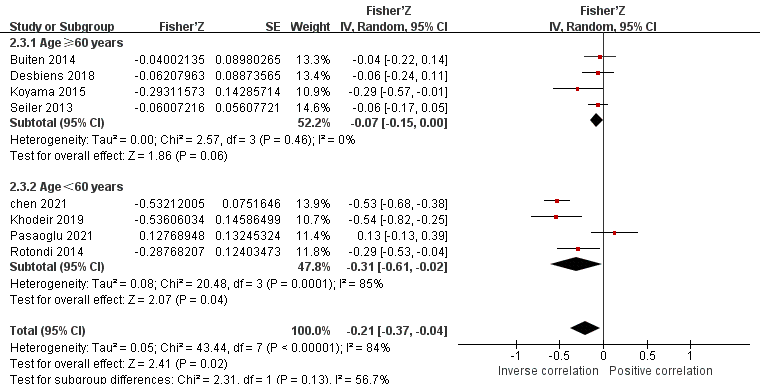


**Supplemental Figure 4c**. Subgroup analysis results of sKlotho level and P on sample size.


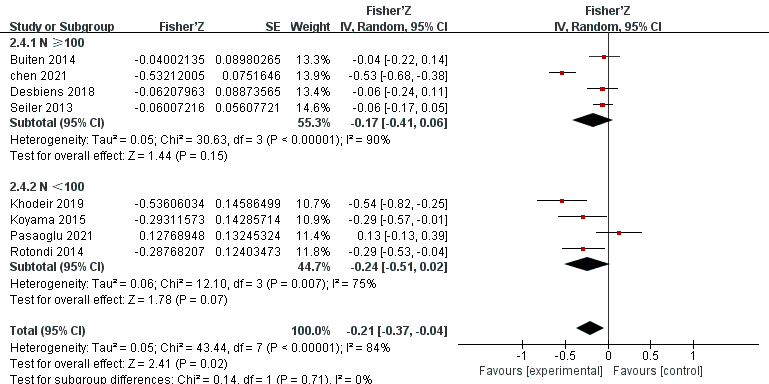


**Supplemental Figure 4d**. Subgroup analysis results of sKlotho level and P on study quality.


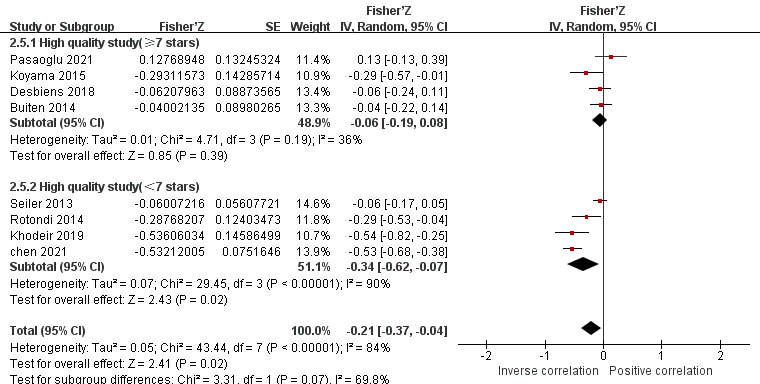


**Supplemental Figure 5**. Funnel plot of sKlotho levels and PTH.


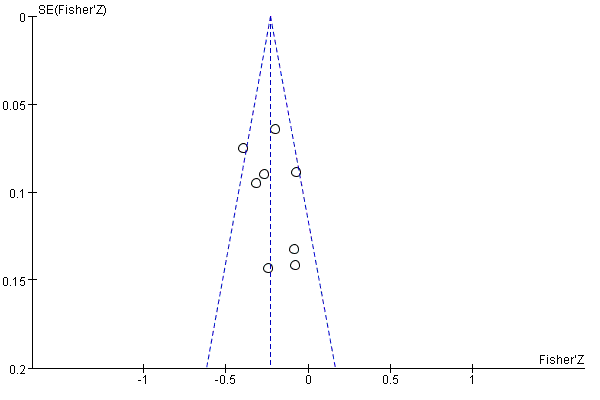


**Supplemental Figure 6a.** Subgroup analysis results of sKlotho level and PTH on disease models.


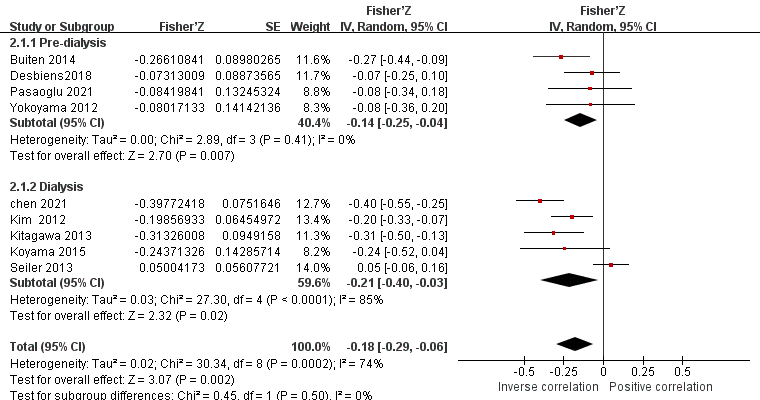


**Supplemental Figure 6b.** Subgroup analysis results of sKlotho level and PTH on age.


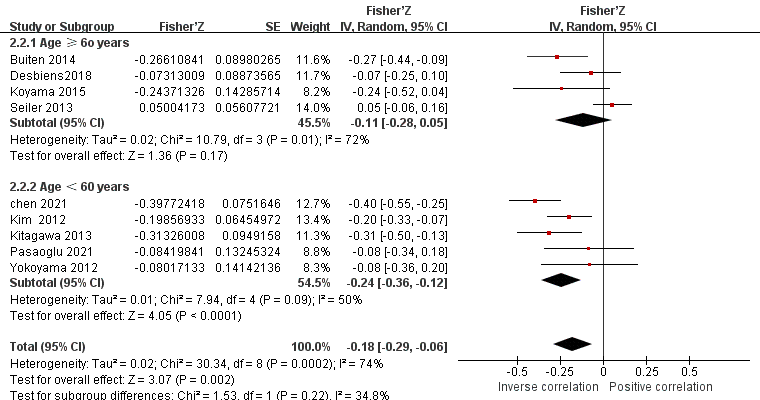


**Supplemental Figure 6c**. Subgroup analysis results of sKlotho level and PTH on sample size.


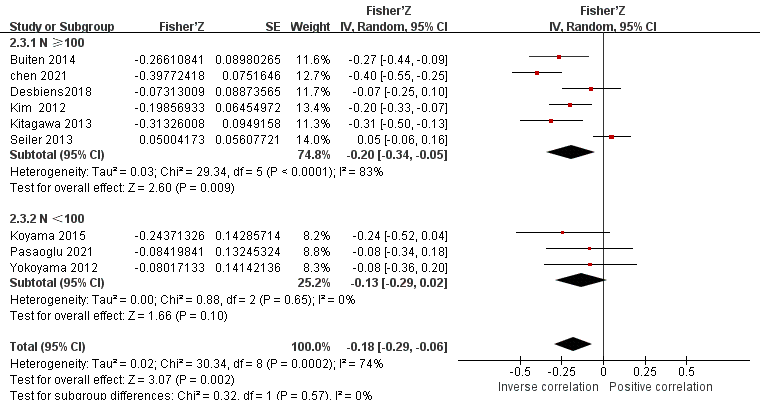


**Supplemental Figure 6d**. Subgroup analysis results of sKlotho level and PTH on study quality.


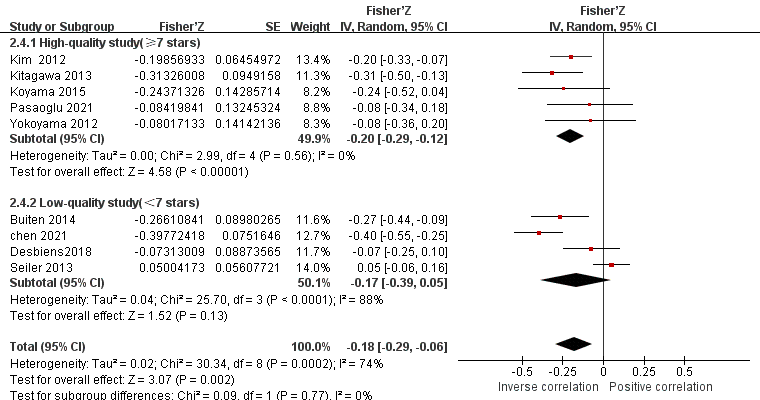


**Supplemental Figure 7**. Funnel plot of sKlotho levels and VC.


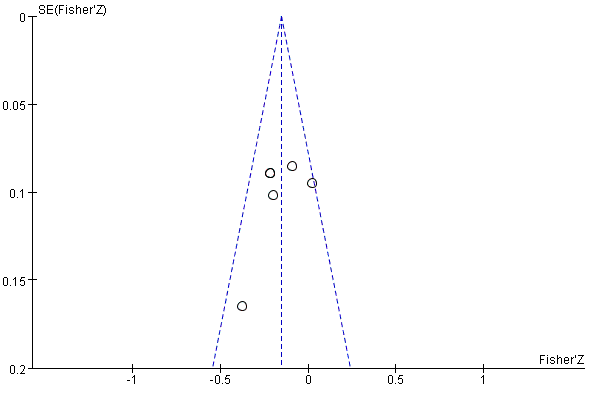

Supplement: Supplementary file 1 — Supplementary Information. [file 41598_2024_54812_MOESM1_ESM.docx]
